# COVID-19 Vaccines in Indonesia: Knowledge, Attitudes, and Acceptance Among Dental Professionals

**DOI:** 10.3389/fmed.2021.784002

**Published:** 2021-12-21

**Authors:** Citra Fragrantia Theodorea, Armelia Sari Widyarman, Iwan Dewanto, Tri Erri Astoeti

**Affiliations:** ^1^Department of Oral Biology, Faculty of Dentistry, Universitas Indonesia, Depok, Indonesia; ^2^Department Head of Microbiology, Faculty of Dentistry, Trisakti University, Jakarta, Indonesia; ^3^Faculty of Medical and Health Science, School of Dentistry, University Muhammadiyah Yogyakarta, Bantul, Indonesia; ^4^Department of Public Health and Preventive Dentistry, Faculty of Dentistry, Trisakti University, Jakarta, Indonesia

**Keywords:** acceptance, COVID-19, knowledge, vaccine, dentist, Indonesia

## Abstract

**Background:** At the time of writing, the number of Coronavirus Disease 2019 (COVID-19) cases in Indonesia has exceeded 2 million. The COVID-19 pandemic has caused severe disruptions in and unprecedented challenges to healthcare systems, including the one in Indonesia. Healthcare professionals, especially dentists, have an increased risk of contracting the virus. Many dental professionals in Indonesia have been exposed to the virus through close contact with aerosols and droplets from the oral cavities of their patients and have subsequently become infected. The Indonesian government commenced its COVID-19 vaccination program in January 2021. It is necessary to achieve high COVID-19 vaccination coverage rates among health workers. However, immunizations are voluntary, and some healthcare workers may be reluctant to receive them. The aim of this study was to evaluate the knowledge, attitudes, and acceptance of dental professionals of COVID-19 vaccines.

**Materials and Methods:** A cross-sectional study was conducted among dentists taking part in the Indonesia Dental Association webinar in June 2021. Google Docs was used to create an online questionnaire, which was disseminated to the participants at the end of the webinar. The questionnaire consisted of 15 questions developed after being reviewed by experts. The questionnaire collected demographic data (age, gender, location, and affiliation/institution) and data on the dental professionals' knowledge and acceptance of COVID-19 vaccines in Indonesia as well as their attitudes toward COVID-19 vaccines. The respondents selected one option (agree/neutral/disagree) in response to each statement in the questionnaire. For data analysis, the respondents were divided into three groups according to their professional activity/employment category: national hospital (GOV), private hospital (PRIVATE), or academic faculty in a dental school (ACADEMIC). The data were analyzed using descriptive statistics and expressed as frequencies and percentages. A chi-square test was used to investigate the association between professional activity and acceptance of COVID-19 vaccines.

**Results:** In total, 779 dentists from 34 provinces in Indonesia completed the questionnaire. There were 646 (83%) females and 133 (17%) males, with an age range of 24–73 years. In terms of the distribution of professional affiliations, the respondents included 23 (3%) academics, 285 (36.5%) private hospital/private practice practitioners, and 471 (60.5%) national hospital practitioners. In the statistical analysis, unadjusted odds ratios (ORs) were calculated with their 95% confidence intervals (CIs).

**Conclusions:** Acceptance of COVID-19 vaccines is an essential determinant of vaccine uptake and the likelihood of controlling the COVID-19 pandemic. There is agreement between dental professionals in private hospitals and academic faculties (dental school) regarding the need for COVID-19 vaccination. Developing strategies to reduce public hesitation and increase trust is vital for implementing vaccination programs, and dentists can play a role in increasing the uptake of COVID-19 vaccines.

## Introduction

SARS-CoV2 is a new virus that infects the respiratory system. It originated in Wuhan, China and was first reported in December 2019. This virus eventually resulted in a worldwide pandemic, and the disease caused by SARS-CoV2 is now called Coronavirus Disease 2019 (COVID-19) (1). Coronavirus Disease 2019 has had devastating effects, with over 200 million confirmed cases of infection and over 4 million deaths as of 11 August 2021 (2). In particular, Indonesia has struggled to contain the infection and maintain the health of its citizens. As of 11 August 2011, there were over 100 thousand deaths, over 3.7 million confirmed cases, and over 3.2 million recovered individuals reported and documented in Indonesia (3).

Direct human-to-human transmission and indirect contact via aerosol droplets are the main transmission methods for COVID-19. The droplets can be suspended in the air for approximately 3 h (4). This has especially affected the efficiency of dental health workers/dentists. Many dental professionals in Indonesia have been exposed to the virus through close contact with aerosols and droplets from the oral cavities of their patients and have subsequently become infected. There is a high possibility that aerosols will be generated during dental procedures. Accordingly, dentists are at a very high risk of COVID-19 infection (5). Although proper usage of personal protective equipment is mandatory to help mitigate the transmission of COVID-19 during dental procedures, the danger of COVID-19 infection as a result of dental procedures remains imminent (6).

For decades, vaccinations have been considered as the best method so far to control rapidly spreading of infectious diseases (7). A vaccine must fulfill at least two requirements before it can be widely used in humans. First, the vaccine must be safe for all population types (age, sex, race). Second, the vaccine must demonstrate acceptable efficacy against the pathogen (8). Recently, COVID-19 vaccines have been produced and distributed in an attempt to minimize the spread of COVID-19 infection and achieve herd immunity to eventually reduce COVID-19 infection and transmission rates (9). Countries around the world are eagerly awaiting the availability of an effective vaccine to combat the spread of COVID-19.

In order to achieve high COVID-19 vaccination coverage rates in public, several companies that produce COVID-19 vaccines, such as BioNTech/Pfizer (9), Oxford-AstraZeneca (10), and Sinovac (11) have been distributed in many countries. Indonesia, first implemented the COVID-19 vaccine broadly in January, 2021 in Jakarta, with the initial target for health workers and public servants. Accordingly, Indonesia has provided Covid-19 vaccines (e.g., Sinovac, AstraZeneca COVAX Facility, and Sinopharm) for free as part of the government's vaccination program to eradicate COVID-19 in massive. Health workers have been put into the first-phase category in the governmental vaccination program, allowing them to immediately receive the vaccine so that they may continue treating patients (12). However, information regarding the vaccines is still poorly understood, even by health workers, including dentists. This is important, as vaccinated dentists can prevent and/or reduce COVID-19 infection during dental procedures, which is crucial since dentists need to continuously perform dental procedures to make a living (13).

Previous studies have documented Indonesian dentists' knowledge about COVID-19 (14, 15). Nevertheless, there has been no research on vaccine acceptance yet from the perspective of Indonesian dentists. Attempting to bridge this knowledge gap, this research aims is to evaluate and assess dental professionals' knowledge, attitudes, and acceptance of COVID-19 vaccines in Indonesia. Hopefully, the information acquired from this research can help inform the Indonesian government's policy-making decisions and improve Indonesian dentists' awareness concerning COVID-19 vaccines.

## Methods

A cross-sectional study was conducted involving dentists taking part in the Indonesia Dental Association's webinar in June 2021. Prior the online questionnaire were distribute, we construct the questions and answer, then reviewed by the expertise of dental public health and statistician to check the realibility and validity of the questionnaire. Ethical approvement was received from the ethical commission review board. Pre-survey was did in small group of target participants. Furthermore, we then continue to distribute to the participants of dentistry webinars after received the informed consent form the participant.

Google Docs was used to create an online questionnaire, which was disseminated to the participants at the end of the webinar. The questionnaire consisted of 15 questions developed after being reviewed by experts. The questionnaire collected demographic data (age, gender, location, and affiliation/institution) and data on the dental professionals' knowledge and acceptance of COVID-19 vaccines in Indonesia as well as their attitudes toward COVID-19 vaccines. The respondents selected one option (agree/neutral/disagree) in response to each statement in the questionnaire.

The sample size was calculated using the G power sample size formula, with a margin of error of 5%, 95% confidence level, 50% distribution response, thus with a population of 35,000 dentists, the required number of samples is a minimum of 380 dentists.

For data analysis, the respondents were divided into three groups according to their professional activity/employment category: national hospital (GOV), private hospital (PRIVATE), or academic faculty in a dental school (ACADEMIC). This study was approved by the Ethical Commission Review Board of the Faculty of Dentistry Trisakti University (no: 518/S1/KEPK/FKG/8/2021).

### Statistical Analysis

The data were analyzed using descriptive statistics and expressed as frequencies and percentages. A chi-square test was used to investigate the risk ratio between professional activity and acceptance of COVID-19 vaccines. IBM SPSS Statistics version 25 (IBM, Armonk, NY, USA) was used for the statistical analyses. The value of *p* < 0.05 was considered statistically significant.

## Results

In total, 779 dentists from 34 provinces in Indonesia completed the questionnaire. There were 646 (83%) females and 133 (17%) males, with an age range of 24–73 years. In terms of the distribution of professional affiliations, the respondents included 23 (3%) academics, 285 (36.5%) private hospital/private practice practitioners, and 471 (60.5%) national hospital practitioners. The demographic of the respondents is shown in Table 1, while the location of the respondents when joined the webinar is shown in Table 2.

**Table 1:** Demographics of the respondents.

**Category**		***n***	**%**
Sex	Female	646	82.93
	Male	133	17.07
Age group	20–30 years	249	31.96
	31–40 years	280	35.94
	41–50 years	140	17.97
	51–60 years	86	11.04
	>60 years	23	2.95
	No information	1	0.13
Division	Academic	23	2.95
	Private	285	36.59
	Government	471	60.46
Status	General practitioner	741	95.12
	Post graduate resident	4	0.51
	Specialist	34	4.36

**Table 2:** Location of the respondents.

**Category**	**Location**	***n***	**%**
Region	Riau Islands	29	3.72
	Bangka Belitung Islands	8	1.03
	Aceh	14	1.80
	North Sumatera	55	7.06
	West Sumatera	24	3.08
	South Sumatera	9	1.16
	Bengkulu	4	0.51
	Jambi	4	0.51
	Lampung	20	2.57
	Banten	39	5.01
	Jakarta	140	17.97
	West Java	110	14.12
	Central Java	59	7.57
	Yogyakarta	23	2.95
	East Java	95	12.20
	Bali	33	4.24
	East Nusa Tenggara	8	1.03
	West Nusa Tenggara	1	0.13
	Central Kalimantan	4	0.51
	North Kalimantan	1	0.13
	South Kalimantan	3	0.39
	West Kalimantan	5	0.64
	East Kalimantan	18	2.31
	South Sulawesi	37	4.75
	Central Sulawesi	3	0.39
	West Sulawesi	2	0.26
	Southeast Sulawesi	10	1.28
	North Sulawesi	6	0.77
	Maluku	1	0.13
	West Papua	9	1.16
	Outside Indonesia	1	0.13
	No information	4	0.51

**Figure 1:**
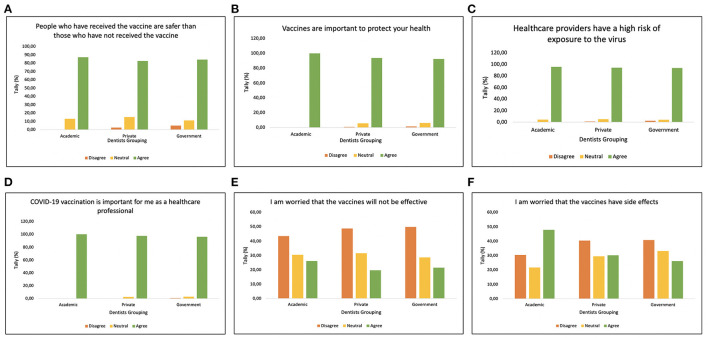
Attitude graph regarding the statement, **(A)** “People who have received the vaccine are safer than those who have not received the vaccine,” **(B)** “Vaccine are important to protect your health,” **(C)** “Healthcare providers have a high risk of exposure to the virus,” **(D)** “COVID-19 vaccination is important for me as a healthcare professional,” **(E)** “I am worried that the vaccines will not be effective,” and **(F)** “I am worried that the vaccines have side effects.”

The respondents' perceptions of the COVID-19 pandemic and vaccination concerns are shown in Figure 1. The majority of dentist in Indonesia agreed that people who have received vaccine are safer than those who have not received vaccine, hence they agreed the importance of vaccine to protect our health (Figures 1A–D). In addition, dental professionals awarded that as a frontline workers, they have a high risk of exposure to the virus, hence COVID-19 vaccination is important. However, the participant's response were varied to the vaccine efficacy and side effect (Figures 1E,F). Only dentist from academic worried that the vaccines have side effect.

**Figure 2:**
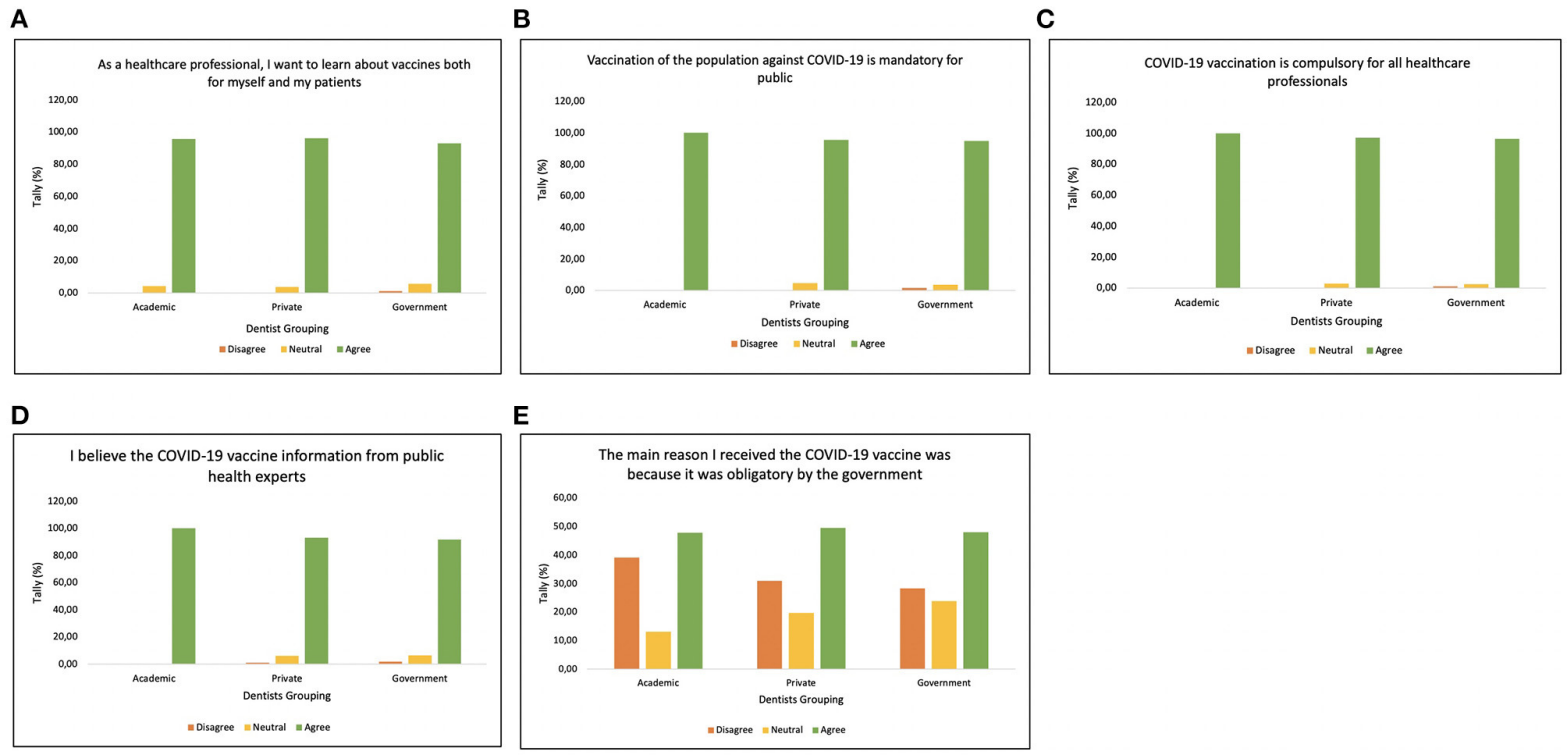
Attitude graph regarding statement, **(A)** “As a healthcare professional, I want to learn about vaccines both for myself and my patients,” **(B)** “Vaccination of the population against COVID-19 is mandatory for public health,” **(C)** “COVID-19 vaccination is compulsory for all healthcare professionals,” **(D)** “I believe the COVID-19 vaccine information from public health experts,” and **(E)** “The main reason I received the COVID-19 vaccine was because it was obligatory by the government.”

Furthermore, their knowledge, attitudes, and acceptance of the COVID-19 vaccine are shown in Figure 2. All the dentist in Indonesia were aware and want to learn about vaccine not only for themselves but also to educate their patients (Figure 2A). In addition, majority of dentist were accept that COVID-19 vaccination is mandatory for healthcare workers and public (Figures 2B,C), thus dentist believe the COVID-19 vaccine information from public health experts (Figure 2D). However, the participant's response were varied in receiving COVID-19 vaccine due to it was obligatory by the government. The statistical analysis, unadjusted odds ratios (ORs) were calculated with their 95% confidence intervals (CIs) as shown in Table 3.

**Table 3:** The statistical analysis, unadjusted odds ratios (ORs) were calculated with their 95% confidence intervals (CIs).

**Survey item**	**Institution**	Agree; neutral ^*^ frequency [%]	**OR (95% CI)**	***P*-value**
People who have received the vaccine are safer than those who have not received the vaccine	Gov	129[28]; 10[25]	1 (Reference)	
	Private	62[22]; 1[0]	0.21 (0.03–1.66)	>0.05
	Academic	3[13]; 1[0]	4.3 (0.41–45.2)	>0.05
As a healthcare professional, I want to learn about vaccines both for myself and my patients	Gov	97[21]; 27[6]	1 (Reference)	
	Private	60[21]; 11[4]	0.7 (0.3–1.4)	>0.05
	Academic	2[8]; 1[4]	1.8 (0.16–20.6)	>0.05
Vaccines are important to stay healthy	Gov	100[21]; 29[6]	1 (Reference)	
	Private	59[21]; 16[5]	0.94 (0.47–1.94)	>0.05
	Academic	4[17]; 1[0]	0.86 (0.09–8.0)	>0.05
Vaccination of the population against COVID-19 is mandatory for public health	Gov	83[17]; 17[4]	1 (Reference)	
	Private	57[20]; 13[5]	1.1 (0.5–2.5)	>0.05
	Academic	3[13]; 0	1.6 (0.16–1.66)	>0.05
COVID-19 vaccination is compulsory for all healthcare professionals	Gov	64[13]; 12[2]	1 (Reference)	
	Private	29[10]; 0	0.18 (0.02–1.5)	>0.05
	Academic	1[4]; 0	5.3 (0.31–91.2)	>0.05
Healthcare providers have a high risk of exposure to the virus	Gov	84[17]; 19[4]	1 (Reference)	
	Private	55[20]; 14[5]	1.1 (0.5–2.4)	>0.05
	Academic	3[13]; 1[4]	1.5 (0.15–15.0)	>0.05
COVID-19 vaccination is important for me as a healthcare professional	Gov	63[13]; 14[3]	1 (Reference)	
	Private	35[13]; 7[2]	0.9 (0.3–2.4)	>0.05
	Academic	3[13]; 0	4.5 (2.7–76.4)	<0.05^*^
I am worried that the vaccines will not be effective	Gov	60[13]; 135[29]	1 (Reference)	
	Private	35[12]; 90[31]	11.9 (5.8–24.3)	<0.05^*^
	Academic	4[17]; 7[30]	8.1 (2.06–31.7)	<0.05^*^
I am worried that the vaccines have side effects	Gov	59[12]; 156[33]	1 (Reference)	
	Private	53[18]; 84[29]	0.6 (0.4–0.9)	>0.05
	Academic	5[22]; 5[21]	0.4 (0.1–1.4)	>0.05
I believe the COVID-19 vaccine information from public health experts	Gov	127[27]; 30[6]	1 (Reference)	
	Private	69[24]; 17[6]	1.0 (0.54–2.0)	>0.05
	Academic	2[9]; 0	2.1 (0.19–24.1)	>0.05
The main reason I received the COVID-19 vaccine was because it was obligatory by the government	Gov	92[20]; 112[23]	1 (Reference)	
	Private	50[18]; 55[20]	0.9 (0.56–1.4)	>0.05
	Academic	3[13]; 3[13]	0.8 (0.16–4.2)	>0.05

* *Participants who responded affirmatively agree/neutral/disagree*.

## Discussion

The current COVID-19 pandemic has resulted in many hardships related to the economy, health, social circumstances, and education. Since COVID-19 is spread through human–human interaction, it is imperative to limit face-to-face interactions to the greatest extent possible to prevent COVID-19 transmission (4). Consequently, people are now attempting to adjust their human interactions in response to the new normal conditions, particularly in Indonesia. For instance, in the economic sector, people have started to use cashless applications for transactions; in the social and education sectors, online platforms have been heavily and forcefully promoted to maintain face-to-face discussion, teaching and learning. In the health sector, the online platforms also have been widely used for webinar, virtual conference, and telemedicine, meanwhile people have adopted healthy lifestyle behaviors, such as wearing masks at all times, physical distancing, supplement consumption, and, ultimately, vaccination to create herd immunity (16, 17).

Vaccination helps the human body to produce the necessary antibodies specific to SARS-CoV2 by recognizing the specific proteins generated by SARS-CoV2, thus reducing the severity of the disease or lowering the chances of COVID-19 infection (18). According to the Indonesian government, vaccination is mandatory for every Indonesian citizen, especially health workers as frontline workers (8). However, even some of healthcare workers might have vaccine hesitancy to receive it based on their personal beliefs.

To the best of the authors' knowledge, this is the first research to assess the level of acceptance of dentists concerning the COVID-19 vaccination in Indonesia. It was deemed necessary to increase the knowledge, perception and attitude of COVID-19 vaccination in Indonesia. Dentists are at high risk of COVID-19 infection. Based on the results of this study, dentists from private, academic, and government clinics agreed that vaccination makes an individual healthier and safer from COVID-19 infection and are aware that dentists have a higher risk of becoming infected by COVID-19. Thus, they believed that dentists should get vaccinated for their own well-being. These findings are supported by the fact that SARS-CoV2 vaccines were made in an attempt to increase human immunity against the virus, thus reducing its severity in the case vaccinated people do become infected by SARS-CoV2 (9). Interestingly, although the majority of Indonesian dentists disagreed with the statement that they were worried about the efficacy and side effects of the vaccines, some agreed that they hesitant about the efficacy of vaccination and worried about its side effects. Indonesian health workers were the first phase target of the governmental vaccination program, and Sinovac was the vaccine used in this phase at that time. The negative attitudes may have been related to the report on Sinovac's clinical trial, which provided conflicting results in some countries (18), which potentially causing confusion and public distrust. In addition, it may also be possible that this vaccine remain offensive to the interplay between religion beliefs, politics, and personal beliefs that influence individual and community decision around vaccination (19).

Many factors could affect vaccine acceptance. Studies have assessed the impact of perceived risk, vaccine efficacy, and job-type employment on vaccine acceptance (20). However, due to cultural diversity, each country might have a different level of acceptance and associated determinants. A previous study revealed that religiosity was an essential determinant influencing parents' approaches to health management issues (19, 21). Thus, the Indonesian government or other related organizations should focus on providing information regarding the benefits of vaccination to the community and ensure that the vaccines are easily accessible (19).

Indonesia, with its 270 million people, has the same problem regarding the vaccination supply. With a target to inoculate 181 million people, Indonesia requires at least 426 million vaccine doses. Although Indonesia reached an agreement with Sinovac, a vaccine produced by a China-based biopharmaceutical company (22), for most of its population, the successful COVID-19 vaccination program requires a robust logistic chain, systematic distribution, and a high level of acceptance of COVID-19 vaccination (23). The vaccination program also requires clear communication and message to the public, including explaining the government's plans and evaluations (24). At the same time, the public demand the transparency about the vaccination program to increase public trust and acceptance of vaccines (25).

Based on the results, the majority of dentists strongly agreed that vaccination should be mandatory for both citizens and health workers to ensure better health conditions in the current pandemic situation, and the dentists were willing to learn more about the vaccines for their patients' well-being. These results match with other findings that showed that COVID-19 vaccination acceptance is important for maintaining patients' well-being, ensuring the continuity of dental health performance by dentists, and reducing the COVID-19 positivity rate (13). Control of COVID-19 through vaccination is not only dependent on vaccine efficacy and safety. Vaccination efforts also rely on the acceptance and awareness of healthcare professionals to successfully control the virus (26).

All of the respondents agreed that they believed vaccination information from public health experts. Learning from experts is one way to reduce misinformation/hoaxes and increases dentists' trust in COVID-19 vaccination (27). Another interesting finding was that the majority of the respondents agreed that they must be vaccinated because it was required by the government, not based on their own willingness. This attitude is probably due to the local government's policy of fining people who resist vaccination. This extreme measure was taken by the government in hopes of speeding up its COVID-19 outbreak management and establishing herd immunity in Indonesia (28). Ideally, the government should work together with the media and public health experts to provide facts about COVID-19 while dispelling myths to prevent the spread of misinformation and improve people's trust in the government (29).

This study has some limitation, First, the participant of this study may not able to a strong association; hence the broader co-workers in dental professional, such as dental nurse need to include in further study. Second, the online questionnaire distribution found difficult to access especially for the elderly participant over 60 years old. Third, comparative study between Indonesia and some other countries especially in South East Asia region may need to obtain the adequate information acceptance of the COVID-19 vaccine.

## Conclusions

The acceptance of COVID-19 vaccines is an essential determinant of vaccine uptake and is crucial for controlling the COVID-19 pandemic. There is an agreement between dental professionals in private hospitals and academic faculties (dental school) regarding the need for COVID-19 vaccination. Developing strategies to reduce public hesitation and increase trust is vital for successfully implementing vaccination programs, and dentists can play a key role in increasing the uptake of COVID-19 vaccines. Hopefully, the results of this study can help to inform the Indonesian government's policy-making regarding accelerating COVID-19 vaccination.

## Data Availability Statement

The raw data supporting the conclusions of this article will be made available by the authors, without undue reservation.

## Ethics Statement

This study was approved by the Ethical Commission Review Board of the Faculty of Dentistry Trisakti University (no: 518/S1/KEPK/FKG/8/2021).

## Author Contributions

CT and AW contributed to the concept, design of the study, and interpreted the results. AW, CT, and ID collected and analyzed the data. AW, CT, ID, and TA wrote sections of the manuscript. All authors contributed to the article and approved the submitted version.

## Funding

This work was supported in part by grants from Universitas Indonesia through grant nos. NKB-0246/UN2.R3.1/HKP.05.00/2019 and BA-375/UN2.RST/PPM.00.03.01/2021 to CT.

## Conflict of Interest

The authors declare that the research was conducted in the absence of any commercial or financial relationships that could be construed as a potential conflict of interest.

## Publisher's Note

All claims expressed in this article are solely those of the authors and do not necessarily represent those of their affiliated organizations, or those of the publisher, the editors and the reviewers. Any product that may be evaluated in this article, or claim that may be made by its manufacturer, is not guaranteed or endorsed by the publisher.
